# Intestinal NSD2 Aggravates Nonalcoholic Steatohepatitis Through Histone Modifications

**DOI:** 10.1002/advs.202402551

**Published:** 2024-06-26

**Authors:** Yijia Zhang, Yuan Qiao, Zecheng Li, Donghai Liu, Qi Jin, Jing Guo, Xin Li, Long Chen, Lihong Liu, Liang Peng

**Affiliations:** ^1^ Beijing Key Laboratory of Bioprocess College of Life Science and Technology Beijing University of Chemical Technology Beijing 100029 P. R. China; ^2^ Beijing Key Laboratory for Immune‐Mediated Inflammatory Diseases Institute of Clinical Medical Sciences China‐Japan Friendship Hospital Beijing 100029 P. R. China

**Keywords:** hepatic steatosis, histone dimethyltransferase, intestinal barrier, tight junction

## Abstract

Mounting clinical evidence suggests that a comprised intestinal barrier contributes to the progression of nonalcoholic steatohepatitis (NASH); nevertheless, the precise mechanism remains elusive. This study unveils a significant upregulation of nuclear receptor‐binding SET domain protein 2 (NSD2) in the intestines of obese humans and mice subjected to a high‐fat cholesterol diet (HFCD). Intestine‐specific NSD2 knockout attenuated the progression of intestinal barrier impairment and NASH, whereas NSD2 overexpression exacerbated this progression. Mechanistically, NSD2 directly regulates the transcriptional activation of *Ern1* by demethylating histone H3 at lysine 36 (H3K36me2), thus activating the ERN1–JNK axis to intensify intestinal barrier impairment and subsequently foster NASH progression. These findings elucidate the crucial role of NSD2‐mediated H3K36me2 in intestinal barrier impairment, suggesting that targeting intestinal NSD2 can represent a novel therapeutic approach for NASH.

## Graphical abstract

Zhang et al. identify intestinal NSD2 as a novel and essential regulator of intestinal barrier and nonalcoholic steatohepatitis (NASH). NSD2 directly regulates the transcriptional activation of *Ern1* through the demethylation of histone H3 at lysine 36 (H3K36me2), resulting in the activation of the ERN1–JNK axis, exacerbation of intestinal barrier impairment, and subsequent promotion of NASH progression.

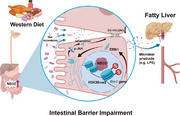

## 1 Introduction

Nonalcoholic fatty liver disease (NAFLD) presently stands as the most prevalent chronic liver condition globally, with an estimated prevalence of 30% among the global adult population.^[^ ^1^, ^2^ ^]^ It encompasses a spectrum of pathology ranging from simple steatosis to nonalcoholic steatohepatitis (NASH).^[^ ^3^ ^]^ Within this spectrum, a subgroup of individuals with NAFLD progresses to NASH, characterized by hepatocellular ballooning and inflammation of the liver lobules, potentially leading to advanced cirrhosis or even hepatocellular carcinoma.^[^ ^4^, ^5^, ^6^ ^]^ Despite numerous studies attempting to unveil the etiology of NASH, approved pharmacotherapies for the disease remain limited. Consequently, delving into the underlying mechanisms of NASH development and progression is imperative to pinpoint potential molecular targets for treatment.

Mounting evidence underscores the significance of the intestine–liver axis in NASH pathogenesis. A compromised intestinal epithelial barrier leads to the translocation of intestinal bacteria, endotoxins, or other bacterial products into the liver, triggering an immunological process that culminates in liver inflammation.^[^ ^6^, ^7^, ^8^ ^]^ Individuals with NASH exhibit heightened intestinal permeability, which correlates with the severity of hepatic steatosis and inflammation.^[^ ^9^ ^]^ Recent research has identified several novel intestinal targets implicated in NASH development,^[^ ^10^, ^11^, ^12^ ^]^ suggesting that genes associated with intestinal epithelial barrier integrity represent potential targets for improving NASH.

The nuclear receptor‐binding SET domain‐containing protein (NSD) family participates in gene regulation, with its members (NSD1, NSD2, and NSD3) featuring SET structural domains endowed with methyltransferase activity. NSD2, also known as MMSET or WHSC1, catalyzes the dimethylation of lysine 36 in histone H3 (H3K36me2).^[^ ^13^ ^]^ Research indicates that H3K36me2 promotes transcript initiation and extension and antagonizes polymerase silencing.^[^ ^14^, ^15^ ^]^ Consequently, NSD2‐mediated H3K36me2 is recognized as a hallmark of actively transcribed genes.^[^ ^16^ ^]^ Beyond H3K36me2, NSD2 represses transcription by generating H3K36me3 or H4K20me3.^[^ ^13^, ^17^ ^]^ Some studies additionally suggest the dimethylation of NSD2 at H4 lysine 20 (H4K20me2).^[^ ^17^, ^18^ ^]^ In humans, heterozygous deletion of NSD2 is associated with Wolf–Hirschhorn syndrome, a developmental disorder characterized by cognitive and developmental abnormalities.^[^ ^19^, ^20^ ^]^ NSD2 dysregulation is further implicated in the pathogenesis of diverse cancers, ranging from hematologic malignancies to solid tumors.^[^ ^21^, ^22^, ^23^ ^]^ In metabolic diseases, the depletion of the H3K36 methyltransferase NSD2 leads to profound whitening of brown adipose tissue (BAT) and insulin resistance in white adipose tissue (WAT).^[^ ^24^ ^]^ However, the specific actions and underlying mechanisms of NSD2 in intestinal barrier function and NASH remain unclear.

In this study, we employed intestine‐specific NSD2 knockout or activation alongside combined analysis of CUT&Tag and transcriptome data to delineate the role and elucidate the mechanisms underlying intestinal NSD2 in NASH development. Our findings revealed that intestinal NSD2 and H3K36me2 were upregulated in obese humans and mice subjected to a high‐fat cholesterol diet (HFCD). Depletion of intestine‐specific NSD2 depletion substantially ameliorated HFCD‐induced intestinal barrier impairment and NASH. Mechanistically, NSD2 directly governed the transcriptional activation of *Ern1* through H3K36me2, thus activating the ERN1–JNK axis to exacerbate intestinal barrier impairment and subsequently promote NASH progression. Importantly, we observed that UNC6934, an inhibitor specific to NSD2 histone modification sites, demonstrated therapeutic efficacy against intestinal barrier impairment and NASH. Our findings suggest that targeting intestinal NSD2 holds promise as an approach for NASH management.

## 2 Results

### 2.1 Intestinal NSD2 and H3K36me2 Levels are Increased in Obese Individuals and HFCD‐fed Mice

**Figure 1:**
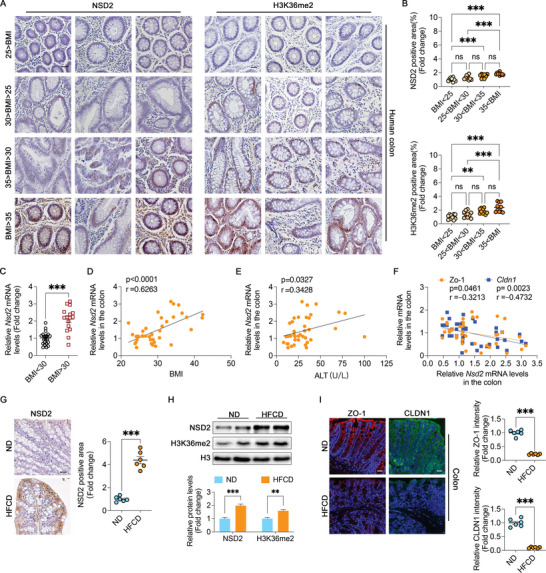
Intestinal NSD2 and H3K36me2 levels are increased in humans with obesity and HFCD‐fed mice. A) IHC staining for the expression of NSD2 (left) and H3K36me2 (right) in human colon biopsies from cohort 1 (*n* = 10 subjects/group). Scale bar, 20 µm. B) The NSD2 and H3K36me2 positive area was quantified by Image J. C) mRNA expression levels of *Nsd2* in human colon biopsies from individuals without obesity (*n* = 23, cohort 2) and with obesity (*n* = 16, cohort 2). D to F) Two‐tailed Pearson's correlation coefficient analysis of colon biopsy *Nsd2* levels with BMI (D), serum ALT level (E), and mRNA levels of colon biopsy *Zo‐1* and *Cldn1* (F), respectively (cohort 2). G) IHC staining for the expression of NSD2 in the colon of indicated mice groups (*n* = 6). Scale bar, 20 µm. H) Representative protein expression levels in the colon of ND‐ or HFCD‐fed mice (*n* = 4). I) Representative immunofluorescence staining of ZO‐1 (red), CLDN1 (green), and DAPI (blue) in mice colon (*n* = 6). Scale bars, 20 µm. The results are presented as means ± SEM, ^*^ *p* < 0.05, ^**^ *p* < 0.01, ^***^ *p* < 0.001. ns means not significant. Statistical analyses were performed by two‐tailed t‐tests between two groups, while one‐way ANOVA and post hoc Bonferroni tests were performed between multiple groups unless otherwise stated.

To explore the potential link between intestinal NSD2 and the intestinal barrier, we assessed NSD2 expression in colon biopsies obtained from nonobese (body mass index [BMI] <30 kg m^−2^) and obese individuals (BMI >30 kg m^−2^) using immunohistochemical (IHC) staining (**Figure** **1**A[left], B). Our findings revealed a significantly elevated NSD2 expression in the obese group compared to that in the nonobese group, with expression levels increasing gradually with BMI. Additionally, intestinal *Nsd2* mRNA levels were higher in the obese group, while the expression profiles of other NSD family members remained unchanged between nonobese or obese groups (Figure 1C; Figure S1A, Supporting Information). Moreover, intestinal *Nsd2* mRNA levels positively correlated with BMI (Figure 1D), serum alanine aminotransferase (ALT; Figure 1E), and triglyceride (TG) levels (Figure S1B, Supporting Information). However, no significant correlation was observed between *Nsd2* mRNA and serum aspartate aminotransferase (AST) levels (Figure S1C, Supporting Information). Furthermore, intestinal *Nsd2* mRNA levels exhibited a negative correlation with intestinal barrier‐related proteins (*Zo‐1* and *Cldn1*; Figure 1F) and a positive correlation with proinflammatory cytokines (*Il‐1α* and *Il1β*; Figure S1D, Supporting Information). Interestingly, IHC staining showed that H3K36me2 expression increased with rising BMI (Figure 1A[right], B). Furthermore, H3K36me3, H4K20me2, and H4K20me3 displayed no significant alterations in protein levels between the nonobese or obese groups (Figure S1E, Supporting Information). These findings suggest that levels of intestinal NSD2 and H3K36me2 levels are elevated in obese individuals.

HFCD exposure compromises the intestinal barrier and triggers NASH development in mice.^[^ ^25^, ^26^ ^]^ We observed upregulation of NSD2 and H3k36me2 expression in the colons of mice fed HFCD for 18 weeks (Figure 1G,H), consistent with clinical samples, while the expression levels of *Nsd1*, *Nsd3*, and other histone proteins potentially modified by NSD2 remained largely unchanged (Figure S1F,G, Supporting Information). Importantly, the elevated *Nsd2* expression was induced by HFCD rather than by the onset of NASH (Figure S1H, Supporting Information). Concurrently, we observed colonic epithelial erosion and inflammation (Figure S1I,J, Supporting Information), terminal ileal epithelial damage, villous atrophy, and crypt distortion (Figure S1K, supporting information), along with altered expression of intestinal barrier‐related proteins (ZO‐1 and CLDN1; Figure 1I) compared to mice on a normal diet (ND). Additionally, an assessment of intestinal permeability using FITC‐labeled dextran (MW4000, FD‐4) indicated enhanced intestinal permeability in HFCD‐fed mice (Figure S1L, Supporting Information). These observations were accompanied by phenotypic correlations between hepatic steatosis and hepatic inflammation (Figure S1M–Q, Supporting Information). Taken together, these findings suggest a potential connection between elevated intestinal NSD2 levels and NASH pathogenesis.

### 2.2 Intestine‐Specific NSD2 Knockout Attenuates NASH Profile

**Figure 2:**
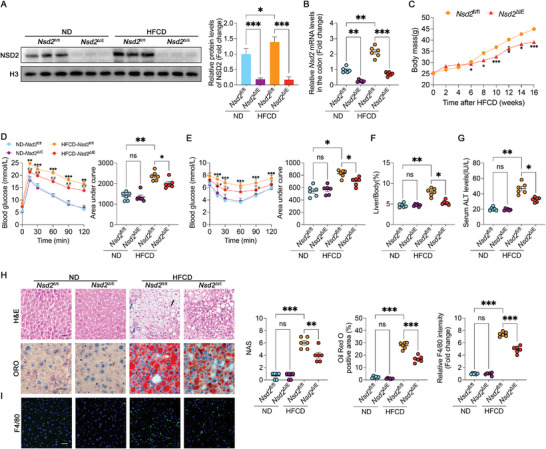
Intestine‐specific NSD2 knockout attenuates NASH profiles. A) Representative protein expression level (*n* = 3) in the colon of indicated mice groups. B) mRNA expression level analysis (*n* = 6) in the colon of indicated mice groups. C) Growth curves of HFCD‐fed *Nsd2* ^fl/fl^ and *Nsd2* ^∆IE^ mice (*n* = 6). D, E) GTT (D) and ITT (E) results and their areas under the curve. F) Liver weight–to–body weight ratios in indicated groups of mice (*n* = 6). G) Serum ALT concentrations in the indicated groups of mice (*n* = 6). H) Representative H&E staining and Oil Red O staining of liver sections. Blue arrows indicate inflammatory cell infiltration and black arrows indicate ballooning degeneration in hepatocytes (*n* = 6). Scale bars, 20 µm. I) Representative immunofluorescence staining of F4/80 (green) and DAPI (blue) in the liver of indicated mice groups (*n* = 6). Scale bars, 20 µm. The results are presented as means ± SEM, ^*^ *p* < 0.05, ^**^ *p* < 0.01, ^***^ *p* < 0.001. ns means not significant. Statistical analyses were performed by two‐tailed t‐tests between 2 groups, while one‐way ANOVA and post hoc Bonferroni tests were performed between multiple groups.

To investigate the role of intestinal NSD2 in NASH progression, an intestine‐specific NSD2 knockout mouse model was generated (Figure S2A, Supporting Information) and confirmed its validity using western blotting, RT‐PCR, and IHC staining (**Figure** **2**A,B; Figure S2B–D, Supporting Information). Subsequently, control mice (*Nsd2* ^fl/fl^) and intestine‐specific NSD2 knockout mice (*Nsd2* ^ΔIE^) underwent an 18‐week treatment regimen with either ND or HFCD. Knockout of intestinal NSD2 led to a reduced weight gain in HFCD‐fed mice without affecting food intake (Figure 2C; Figure S2E,F, Supporting Information). Additionally, results from the glucose tolerance test (GTT) and insulin tolerance test (ITT) indicated that intestine‐specific NSD2 knockout ameliorated HFCD‐induced glucose intolerance and insulin resistance (Figure 2D,E).

Meanwhile, *Nsd2* ^ΔIE^ mice exhibited lower liver‐to‐body weight ratio and serum levels of ALT and AST compared to *Nsd2* ^fl/fl^ mice (Figure 2F,G; Figure S2G, Supporting Information). Subsequent evaluation of lipid deposition and inflammatory cell infiltration in the mice liver tissue revealed that NSD2 knockout significantly attenuated the infiltration of inflammatory cells, hepatosteatosis, and fibrosis in HFCD‐fed mice as evidenced by hematoxylin and eosin (H&E), Oil Red O (ORO) staining and Sirius red staining (Figure 2H; Figure S2H, Supporting Information). Additionally, the accumulation of F4/80‐positive cells was catabatic in the liver of *Nsd2* ^ΔIE^ mice (Figure 2I), accompanied by reduced mRNA levels of adipogenic genes (*Fas* and *Scd‐1*) and proinflammatory cytokines (*Il‐1β*, *Il‐6*, and *Tnf‐α*) in the liver tissue of *Nsd2* ^ΔIE^ mice (Figure S2I, Supporting Information). Thus, our data support the notion that intestine‐specific NSD2 knockout protects mice from HFCD‐induced hepatic steatosis and inflammation.

### 2.3 Intestine‐Specific NSD2 Knockout Protects Mice from HFCD‐Induced Intestinal Epithelial Barrier Impairment

**Figure 3:**
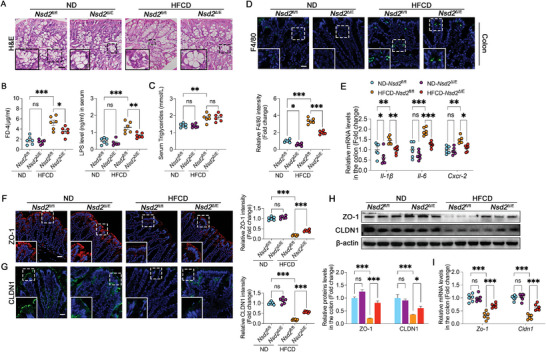
Intestine‐specific knockout protects mice from HFCD‐induced intestinal epithelial barrier impairment. A) H&E staining of the colon from *Nsd2* ^fl/fl^ and *Nsd2* ^∆IE^ mice fed HFCD or ND. B) The serum levels of FD4 (left) and LPS (right) of *Nsd2* ^fl/fl^ and *Nsd2* ^∆IE^ mice fed HFCD or ND (*n* = 6). C) The serum triglyceride levels of *Nsd2* ^fl/fl^ and *Nsd2* ^∆IE^ mice fed HFCD or ND (*n* = 6). D) Representative immunofluorescence staining of F4/80 (green) and DAPI (blue) in the colon of indicated mice groups (*n* = 6). Scale bars, 20 µm. E) Relative mRNA levels of proinflammatory cytokines (*Il‐1β*, *Il‐6*, and *Cxcr‐2*) in the colon of *Nsd2* ^fl/fl^ and *Nsd2* ^∆IE^ mice after HFCD or ND (n = 6). F, G) Representative immunofluorescence staining of ZO‐1 (red, F), CLDN1 (green, G), and DAPI (blue) in mice colon (*n* = 6). Scale bars, 20 µm. H) Representative protein expression levels in the colon of indicated mice groups (*n* = 3). I) Relative mRNA levels of the intestinal barrier (*Zo‐1* and *Cldn1*) in the colon of *Nsd2* ^fl/fl^ and *Nsd2* ^∆IE^ mice fed HFCD or ND (n = 6). The results are presented as means ± SEM, ^*^ *p* < 0.05, ^**^ *p* < 0.01, ^***^ *p* < 0.001. ns means not significant. Statistical analyses were performed one‐way ANOVA and post hoc Bonferroni tests.

The intestinal morphology of *Nsd2* ^ΔIE^ mice was further examined. Intestine‐specific NSD2 knockout ameliorated HFCD‐induced villus atrophy (Figure S3A,B, Supporting Information) and crypt deformation (**Figure** **3**A). Serum FD‐4 and lipopolysaccharide (LPS) levels, reflecting intestinal permeability, were measured, revealing lower levels in *Nsd2* ^ΔIE^ mice (Figure 3B) without abnormal lipid absorption (Figure 3C). The intestine‐specific NSD2 knockout mitigated HFCD‐induced inflammatory cell infiltration in mice colon tissues (Figure 3D) and alleviated the upregulation of proinflammatory cytokine mRNA levels (Figure 3E). More importantly, the protein and mRNA expression levels of a subset of intestinal barrier‐related proteins in the colon of *Nsd2* ^ΔIE^ mice recovered compared with those of *Nsd2* ^fl/fl^ mice (Figure 3F–I; Figure S3C, Supporting Information), suggesting that intestine‐specific NSD2 knockout mitigates HFCD‐induced intestinal barrier impairment. Furthermore, no significant abnormalities were detected in fecal output, energy absorption, and expression levels of lipid absorption‐related genes in *Nsd2* ^∆IE^ mice (Figure S3D–F, Supporting Information), providing additional evidence that the alleviation of NASH resulting from NSD2 knockout occurs primarily through the amelioration of intestinal barrier impairment, rather than affecting intestinal energy and lipid absorption.

### 2.4 NSD2 Deficiency Dampens the ERN1–JNK Axis in the Intestine of HFCD‐fed Mice

**Figure 4:**
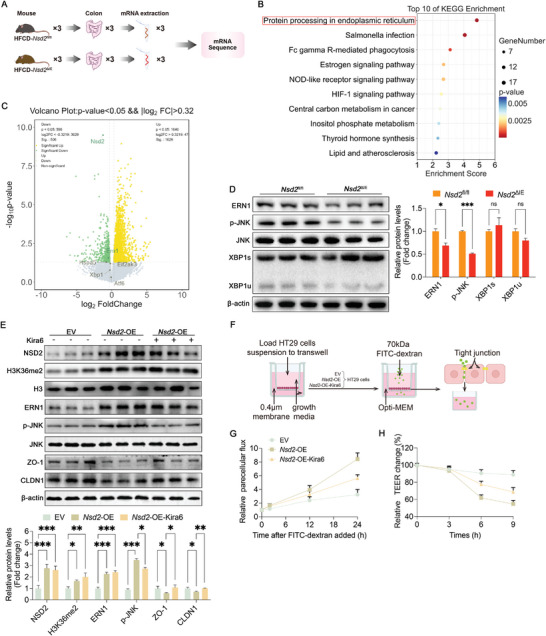
NSD2 deficiency dampens the ERN1–JNK axis in the intestine of HFCD‐fed mice. A) Workflow of RNA‐seq and subsequent mRNA splicing analysis for *Nsd2* ^fl/fl^ and *Nsd2* ^ΔIE^ mice colon fed HFCD. B) KEGG analyses show the altered pathways after NSD2 knockout. C) Volcano plot of NSD2 knockout‐induced transcriptome signature. ER stress sensors were labeled. D) Representative protein expression levels in the colon of *Nsd2* ^fl/fl^ and *Nsd2* ^∆IE^ mice (*n* = 3). E) Representative protein expression levels in the HT29 cells (*n* = 3). F, G) Workflow (F) and quantitative analysis (G) for epithelial FITC‐dextran leakage assay in three groups of HT29 cells, EV, *Nsd2*‐OE, and *Nsd2*‐OE‐Kira6. H) Relative transendothelial electrical resistance (TEER) changes in HT29 cells at different time periods. The results are presented as means ± SEM, ^*^ *p* < 0.05, ^**^ *p* < 0.01, ^***^ *p* < 0.001. ns means not significant. Statistical analyses were performed by two‐tailed t‐tests between two groups, while one‐way ANOVA and post hoc Bonferroni tests were performed between multiple groups.

To delve deeper into the molecular mechanism underlying NSD2's regulation of intestinal epithelial homeostasis, transcriptomic analysis was conducted on the colon samples from *Nsd2* ^fl/fl^ and *Nsd2* ^ΔIE^ mice fed HFCD for 18 weeks (**Figure** **4**A). Among the 19423 expressed genes in mice, 506 were significantly downregulated upon NSD2 knockout (*p* < 0.05, fold change >1.25). Kyoto Encyclopedia of Genes and Genomes pathway analysis revealed significant enrichment of genes associated with protein folding in the endoplasmic reticulum ER (Figure 4B). Similarly, Gene Ontology (GO) term analysis revealed that the enrichment of these downregulated genes related to ER stress (Figure S4A, Supporting Information). This suggests that NSD2 knockout‐induced recovery of the intestinal barrier may be involved in the inhibition of the ER stress pathway, known to contribute to intestinal barrier damage.^[^ ^27^, ^28^, ^29^ ^]^ The volcano plot highlighted that among the ER stress sensor genes (*Ern1*, *Eif2ak3*, *Hspa5, Atf6*, and *Xbp1*), only *Ern1* exhibited significant downregulation in the colon of *Nsd2* ^ΔIE^ mice (Figure 4C), a conclusion further supported by subsequent RT‐PCR analysis (Figure S4B, Supporting Information). Further clinical biopsies revealed that the expression of ERN1 increased with patient BMI (Figure S4C, Supporting Information). Additionally, mRNA expression levels of *Ern1* and *Nsd2* showed a positive correlation in the colonic tissues of the patients (Figure S4D, Supporting Information) indicating a potential link between NSD2 and ERN1 expression. While ERN1 can mitigate ER stress through its endonuclease activity by alternatively splicing *Xbp1* mRNA,^[^ ^30^ ^]^ it can also phosphorylate JNK, thereby activating the JNK signaling pathway‐mediated inflammatory response.^[^ ^31^ ^]^ Protein levels of ERN1 and p‐JNK were significantly reduced in the colon of *Nsd2* ^ΔIE^ mice, but not XBP1 (Figure 4D). Furthermore, the biological processes identified from GO term analysis included the pathway of positive regulation of Jun kinase activity (Figure S4A, Supporting Information). In summary, we propose that NSD2 aggravates inflammatory response and intestinal barrier impairment by upregulating the ERN1–JNK axis.

To validate this hypothesis, HT29 cells were transfected with a NSD2 overexpression plasmid. As expected, NSD2 overexpression led to increased protein levels of ERN1 and p‐JNK (Figure 4E). Moreover, NSD2 overexpression suppressed the expression of intestinal barrier‐related proteins (ZO‐1 and CLDN1; Figure 4E; Figure S4E, Supporting Information). However, treatment with the ERN1 kinase inhibitor Kira6 counteracted the inhibitory effect of NSD2 on intestinal barrier‐related proteins (Figure 4E; Figure S4E, Supporting Information), indicating that NSD2 induces intestinal barrier impairment via the ERN1–JNK axis. This conclusion was further supported by epithelial FITC‐dextran leakage and transendothelial electrical resistance (TEER) measurements performed using Transwell (Figure 4F–H). To delve deeper into the relationship between NSD2 and ERN1, NSD2 knockdown plasmids (sh‐*Nsd2*) were transfected into HT29 cells. Immunofluorescence staining of the cell slides confirmed the knockdown effect of different plasmids on NSD2 in HT29 cells, with successful knockdown and plasmids failure, and subsequent protein expression recovery in the cells observed after transfection for >96 h (Figure S4F, Supporting Information). As NSD2 expression decreased, the expression levels of ERN1 and p‐JNK decreased as well (Figure S4G, Supporting Information). Additionally, levels of proinflammatory cytokines such as *Il‐1β* and *Il‐6* in HT29 cells decreased with NSD2 knockdown (Figure S4H, Supporting Information). Together, these data illustrate that NSD2 disrupts the intestinal barrier by activating the ERN1–JNK axis and the subsequent inflammatory responses.

### 2.5 Histone Methyltransferase Activity of NSD2 Mediates the Upregulation of ERN1

**Figure 5:**
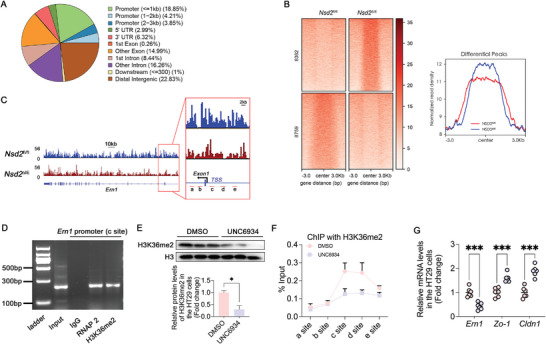
Histone methyltransferase activity of NSD2 mediates the upregulation of ERN1. A) CUT&Tag analysis of colon from *Nsd2* ^fl/fl^ mice and H3K36me2 could be enriched in the promoter region of numerous genes. B) Heatmaps of H3K36me2 CUT&Tag signals in *Nsd2* ^fl/fl^ and *Nsd2* ^∆IE^ mice. The right panel shows the quantitation of the read density about differential peaks between *Nsd2* ^fl/fl^ and *Nsd2* ^∆IE^ mice. C) H3K36me2 peaks in the promoter regions of *Ern1*. Primers targeting different fragments of the *Ern1* gene are indicated, and the c and d sites are H3K36me2 peaks of the *Ern1* promoter region in CUT&Tag. D) ChIP‐PCR analysis to detect H3K36me2 binding in the *Ern1* promoter region. E) Representative protein expression level of H3K36me2 in HT29 cells treated with or without UNC6934 (*n* = 3). F) ChIP‐qPCR was used to detect the binding status of H3K36me2 in the *Ern1* promoter region in HT29 cells treated with or without UNC6934 (*n* = 3). G) Relative mRNA expression levels analysis (*n* = 6) in HT29 cells treated with or without UNC6934. The results are presented as means ± SEM, ^*^ *p* < 0.05, ^**^ *p* < 0.01, ^***^ *p* < 0.001. ns means not significant. Statistical analyses were performed by two‐tailed t‐tests.

The potential mechanism through which NSD2 upregulates ERN1 expression was further investigated. Colon tissues from *Nsd2* ^fl/fl^ and *Nsd2* ^ΔIE^ mice underwent CUT&Tag assay using an anti‐H3K36me2 antibody. The data revealed enrichment of H3K36me2 in the promoter and upstream regions of genes (**Figure** **5**A), suggesting a potential role of NSD2 in H3K36me2 in these regions. When comparing H3K36me2 profiles in colon tissues of *Nsd2* ^fl/fl^ and *Nsd2* ^ΔIE^ mice, the binding peaks of 6569 genes showed no significant difference. However, the binding peaks of 13168 genes were reduced after NSD2 knockout, with 8759 genes exhibiting significantly reduced binding peaks (│logFC│>0.5, *p*‐value < 0.05; Figure 5B). Notably, *Ern1*, a critical gene for ER stress and the JNK pathway was listed among the 8759 genes, and direct occupancy of H3K36me2 within its gene locus was observed in the Integrative Genomics Viewer. Moreover, Integrative Genomics Viewer confirmed the reduction of H3K36me2 modification in the *Ern1* gene promoter and its nearby region in the colon of *Nsd2* ^ΔIE^ mice (Figure 5C). Chromatin immunoprecipitation (ChIP) further verified the binding of H3K36me2 to the *Ern1* gene promoter region in the colon of wild‐type mice fed ND (Figure 5D, Figure S5A, Supporting Information).

Subsequently, HT29 cells were treated with UNC6934 (UNC), a potent NSD2 inhibitor that binds to the PWWP1 domain of NSD2, disrupting the binding of NSD2 to H3K36 and rendering the SET domain of NSD2 nonfunctional.^[^ ^32^ ^]^ UNC treatment attenuated H3K36me2 signaling (Figure 5E), and ChIP‐qPCR showed reduced H3K36me2 recruitment to the *Ern1* promoter region was also reduced (Figure 5F). Consistent with previous results, the expression levels of ERN1 and p‐JNK in UNC‐treated HT29 cells were lower, whereas the expression levels of intestinal barrier‐related proteins (ZO‐1 and CLDN1) were increased (Figure 5G; Figure S5B, Supporting Information). The same results were obtained by epithelial FITC‐dextran leakage assay (Figure S5C, Supporting Information). These data collectively indicate that NSD2 directly regulates the transcriptional activation of *Ern1* through H3K36me2, thereby activating the ERN1–JNK axis to impair the intestinal barrier.

### 2.6 Intestine‐Specific NSD2 Overexpression Aggravated the HFCD‐Induced NASH Phenotype Through Histone Modifications

**Figure 6:**
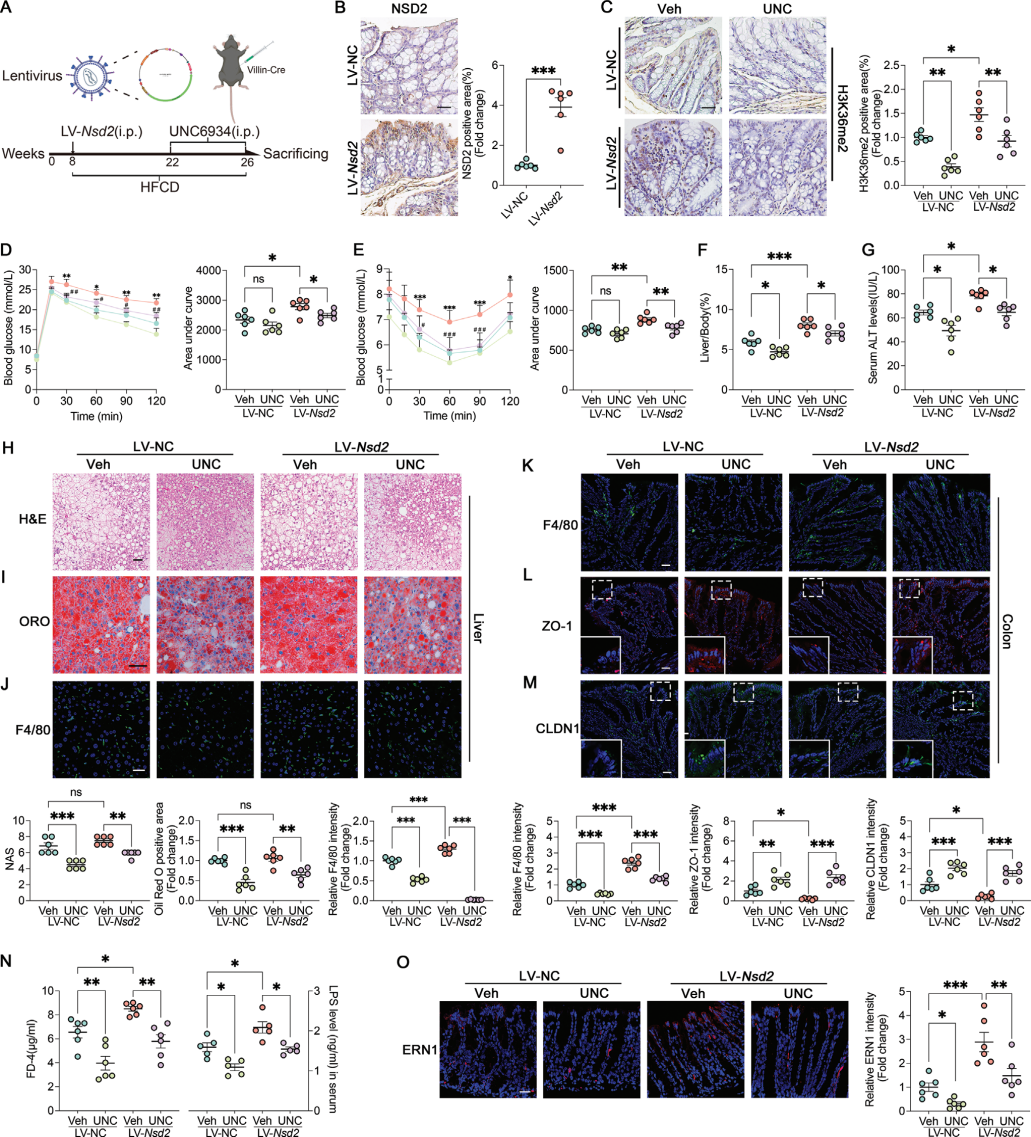
Intestine‐specific NSD2 overexpression aggravated the HFCD‐induced NASH phenotype through histone modifications. A) Construction process of intestine‐specific NSD2 overexpression mice and administration of UNC6934 or Veh (*n* = 6). B,C) Representative IHC staining for the expression of NSD2 in the colon from mice treated with or without LV‐*Nsd2* (B, *n* = 6), and H3K36me2 in the colon from mice treated with or without UNC6934 (C, *n* = 6). Scale bar, 20 µm. D,E) GTT (D) and ITT (E) results and their areas under the curve. F) Liver weight–to–body weight ratios in indicated groups of mice (*n* = 6). G) Serum ALT concentrations in the indicated groups of mice (*n* = 6). H,I) Representative H&E staining (H) and Oil Red O staining (I) of liver sections in the indicated groups of mice (*n* = 6). Scale bars, 50 µm. J) Representative immunofluorescence staining of F4/80 (green) and DAPI (blue) in the liver of indicated mice groups (n = 6). Scale bars, 20 µm. K) Representative immunofluorescence staining of F4/80 (green) and DAPI (blue) in the colon of indicated mice groups (*n* = 6). Scale bars, 20 µm. L,M) Representative immunofluorescence staining of ZO‐1 (red, L) and CLDN1 (green, M) in mice colon (*n* = 6). Scale bars, 20 µm. N) The serum levels of FD4 (left) and LPS (right) in the indicated groups of mice (*n* = 6). O) Representative immunofluorescence staining of ERN1 (red) and DAPI (blue) in the colon of indicated mice groups (*n* = 6). Scale bars, 20 µm. The results are presented as means ± SEM, ^*^ *p* < 0.05, ^**^ *p* < 0.01, ^***^ *p* < 0.001. ns means not significant. Statistical analyses were performed by two‐tailed t‐tests between two groups, while one‐way ANOVA and post hoc Bonferroni tests were performed between multiple groups.

Following this, lentiviruses (LV‐*Nsd2* or LV‐NC) were introduced to construct a mouse model of intestine‐specific NSD2 overexpression (Figure S6A Supporting Information). These mice were subjected to an HFCD diet for 18 weeks. Vehicle (Veh) or UNC was administered daily for 4 weeks before the conclusion of the experiment (**Figure** **6**A). As anticipated, intestinal NSD2 expression was heightened in LV‐*Nsd2* mice, while UNC treatment effectively reduced intestinal H3K36me2 expression (Figure 6B, C; Figure S6B, Supporting Information). Intestine‐specific NSD2 overexpression exacerbated HFCD‐induced metabolic abnormalities, hepatic impairment, insulin resistance, and intestinal barrier impairment in LV‐*Nsd2* mice compared to LV‐NC mice (Figure 6D–N; Figure S6C–E, Supporting Information), whereas these detrimental effects were partially mediated by UNC treatment (Figure 6D–N; Figure S6C–E, Supporting Information). Moreover, UNC treatment effectively dampened the activation of the ERN1–JNK axis induced by NSD2 overexpression (Figure 6O; Figure S6F, Supporting Information). In summary, these data suggest that intestinal NSD2‐driven intestinal barrier impairment relies on MTase activity.

## 3 Discussion

The intestine–liver axis has garnered considerable attention, particularly concerning NASH pathogenesis. Studies have shown that the composition of gut microbiota in patients with NASH differs from that in healthy individuals. They suggest that obesity, a high‐fat diet, and other lifestyle factors can alter the composition of the gut microbiota, leading to imbalances,^[^ ^33^, ^34^, ^35^ ^]^ which in turn can induce the production of intestinal endotoxins, provoke inflammatory responses in the intestinal mucosa, and cause liver damage.^[^ ^36^ ^]^ On the other hand, it has been observed that NASH patients have higher intestinal permeability, which may result in the entry of intestinal endotoxins (lipopolysaccharides, LPS) or other bacterial products in the liver through the intestinal barrier and trigger an inflammatory response. Despite some crucial advances in understanding the role of the intestine–liver axis in NASH, further research is indispensable to comprehensively grasp the underlying mechanisms and develop relevant therapeutic strategies. In this study, we identified NSD2, a histone methylation transferase, as capable of impairing the intestinal barrier through an ERN1–JNK pathway‐mediated inflammatory response, thereby promoting NASH progression.

In this study, a significant elevation in NSD2 expression in the colon tissues of the obese group was observed, with its expression positively correlating with obesity‐related indices and hepatic toxicity, while exhibiting a significant negative correlation with indices associated with intestinal barrier integrity. Additionally, a notable increase in H3K36me2 levels was noted in the colons of both obese and HFCD‐fed mice, suggesting activation of the intestinal NSD2–H3K36me2 axis is activated during obesity, which may play a role in NASH progression. Furthermore, intestine‐specific NSD2 knockout attenuated HFCD‐induced intestinal barrier impairment and NASH. To elucidate the mechanism underlying colonic NSD2's effect on NASH, RNA‐sequencing was performed on colon tissues from HFCD‐fed *Nsd2* ^fl/fl^ and *Nsd2* ^ΔIE^ mice. The sequencing results, combined with phenotypic studies suggested that intestinal NSD2 influences intestinal inflammation and barrier integrity by regulating ERN1 and its downstream effector, JNK. Additionally, employing ChIP technology, we utilized CUT&Tag, a relatively new technique, to validate from multiple perspectives that dimethylated NSD2‐modified H3K36 binds to the *Ern1* promoter region and induces its transcriptional activation. Furthermore, we discovered that a small‐molecule inhibitor, UNC6934, inhibited the histone modification of NSD2, thereby blocking the ERN1–JNK axis and alleviating NSD2‐induced intestinal barrier impairment and NASH progression in HFCD‐fed mice.

As the most statistically significant pathway in transcriptomics, we considered ERN1, involved in protein processing in the ER, to play a major role in intestinal barrier impairment during NASH pathogenesis. However, pathways downregulated in the intestinal tissues of *Nsd2* ^∆IE^ mice also include Salmonella infection, which regulates LPS modification and synthesis triggering intracellular inflammatory responses and apoptotic pathways in the host cell,^[^ ^37^, ^38^, ^39^ ^]^ which in turn regulate intestinal barrier integrity and NASH pathogenesis. Additionally, the NOD‐like receptor signaling pathways induce the release of inflammatory factors, apoptosis, and autophagy, contributing to the intestinal barrier integrity and NASH pathogenesis.^[^ ^40^, ^41^ ^]^ We do not exclude the possibility that these less significant pathways may also play a role in NASH progression alongside ERN1.

NSD family members are characterized by large domain‐containing proteins featuring a conserved SET catalytic domain, two PWWP domains, and five PHD zinc fingers.^[^ ^42^ ^]^ The SET domain comprises three parts: pre‐SET (also known as AWS), SET, and post‐SET domains^[^ ^43^, ^44^ ^]^ responsible for catalyzing histone methylation modifications. These two PWWP domains recognize and bind to histone H3 and DNA, which must be modified.^[^ ^45^ ^]^ The five PHD zinc fingers are crucial for the interactions with other histone methylation proteins.^[^ ^46^, ^47^ ^]^ Despite being the shortest member of the NSD family, NSD2 exhibits a complex and diverse expression pattern.^[^ ^42^ ^]^ NSD2 has three isoforms: NSD2‐long (containing 1365 amino acids), NSD2‐short (containing 647 amino acids), and interleukin‐5 response element II‐binding protein (RE‐IIBP, containing 584 amino acids).^[^ ^48^ ^]^ The NSD2 isoform studied in our article is NSD2‐long, the most common transcript of the *Nsd2* gene, and the only one with histone methyltransferase activity.^[^ ^42^ ^]^

NSD2, as a vital epigenetic modifier, can catalyze the methyl transfer reaction and transfer the methyl group in S‐adenosylmethionine to the Lys36 site on histone H3, forming an H3K36me2/3 epigenetic mark.^[^ ^16^, ^49^ ^]^ This process influences various steps of gene transcription, including initiation, elongation, and splicing, and thus affects biological processes, such as cell metabolism, inflammation, differentiation, development, and apoptosis.^[^ ^16^ ^]^ The role of NSD2's methylation modification function in disease occurrence and progression is intricate and may contribute to various diseases. Numerous studies have linked NSD2 to various cancers, encompassing hematological malignancies and solid tumors.^[^ ^50^, ^51^ ^]^ Moreover, NSD2 plays a crucial role in the normal physiological processes during embryonic development and postnatal life, and complete NSD2 knockout throughout the body may result in severe developmental defects and physiological disorders, potentially leading to embryonic death or early mortality.^[^ ^49^, ^52^ ^]^ While NSD2 depletion in adipose tissue induces significant albinism in BAT and insulin resistance in WAT,^[^ ^24^ ^]^ the presence or absence of an association between NSD2 and intestine‐related or other metabolic disorders has been seldom reported. With the discovery of a new mechanism involving NSD2‐induced damage to the intestinal barrier, this study fills the gap in understanding the role of NSD2 in the intestine‐liver axis.

Enzyme‐mediated post‐translational histone modifications play a pivotal role in providing epigenetic information that governs chromatin conformation and gene transcription, with significant implications for intestinal barrier integrity and hepatic steatosis. Within the intestinal barrier, G9a, which catalyzes the dimethylation of histone H3 lysine 9, modulates the cholesterol biosynthesis pathway in T‐cells and influences their differentiation. Pharmacological inhibition or G9a knockdown alleviates T cell‐associated colitis in mice.^[^ ^53^ ^]^ Another histone methyltransferase SETDB1, mediates the trimethylation of histone H3 at lysine 9, and its deletion disrupts the epithelial barrier, promoting intestinal inflammation through proinflammatory necrotic apoptosis.^[^ ^54^ ^]^ Additionally, SETD2 regulates the transcription of target genes such as *Il1rl1* by modulating the activity of promoters and in‐gene enhancers normally deposited by H3K36me3, thereby maintaining the autostability of T‐regulatory cells in the mouse intestine and gut‐associated lymphoid tissues. This, in turn, suppresses intestinal inflammatory diseases.^[^ ^55^ ^]^ In the liver, HDAC1/2 is recruited by METTL3 to the promoter regions of *Cd36* and *Ccl2*, which facilitates the deacetylation of H3K9 and H3K27. This process inhibits the transcription of *Cd36* and *Ccl2*, thereby suppressing NASH development.^[^ ^56^ ^]^ Additionally, JMJD3 epigenetically upregulates the genes involved in the hepatic autophagy network, including *Tfeb*, *Atg7*, *Atgl*, and *Fgf21*, through histone H3K27me3 demethylation, leading to autophagy‐mediated lipid degradation.^[^ ^57^ ^]^ While the effects of histone modifications on the intestinal barrier or hepatic steatosis involve multiple pathways, there has been no study to date that has explored the relationship between NSD2, H3K36me2, intestinal barrier integrity, and NASH.

As a histone methyltransferase, NSD2 plays a role in multiple cellular processes, such as chromatin modification and gene expression regulation. Its complexity and multifunctionality have prompted researchers to focus on elucidating the downstream pathogenic mechanisms of NSD2, while the upstream regulatory mechanisms have received comparatively little attention and are somewhat uncertain. The mechanism of NSD2 upregulation varies significantly among diseases. The aberrant increase in NSD2 expression observed in patients with multiple myeloma is attributed to the t(4,14) translocation,^[^ ^58^, ^59^ ^]^ while NSD2 methyltransferase hyperactivity in pediatric patients with acute lymphoblastic leukemia is the result of the substitution of glutamic acid with lysine at residue 1099 in its SET structural domain (p. Glu1099Lys and p. E1099K), which in turn leads to tumorigenesis and progression.^[^ ^60^, ^61^, ^62^ ^]^ In addition, NSD2 upregulation involves chromatin remodeling, aberrant regulation of transcription factors, and abnormal histone modifications. Recent studies have shown that the gut microbiota can regulate histone methyltransferases.^[^ ^63^ ^]^ However, no studies have yet reported on the regulatory mechanism of intestinal NSD2. Our study only highlighted the downstream mechanism of NSD2 that leads to intestinal barrier damage and NASH progression. More research and technical tools are needed to understand the mechanism of HFCD‐induced intestinal NSD2 upregulation.

In Figure 1 and Figure S1 (Supporting Information), we observed that H3K36me2 levels in the intestine increased with NSD2 expression, while other histone modifications showed no change in expression. To identify factors or signaling pathways more directly affected by NSD2, we focused our subsequent transcriptomic analysis solely on genes with reduced expression following *Nsd2* knockout. Fortunately, by combining CUT&Tag, we identified the *Ern1* gene, which is directly regulated by NSD2‐mediated dimethylation of H3K36.

Depending on the target domain, small‐molecule NSD2 inhibitors can be categorized as inhibitors that target the SET structural domain catalyzed by NSD2 (NSD2‐SET), inhibitors that target the PHD structural domain of NSD2 (NSD2‐PHD), or inhibitors that target the PWWP1 structural domain (NSD2‐PWWP1).^[^ ^42^ ^]^ Initially, because of the pronounced conservation of catalytic SET structural domains across all histone lysine methyltransferases (HMTases), particularly within the NSD family members (NSD1, NSD2, and NSD3), most inhibitors directed at SET structural domains are commonly identified as pan‐NSD inhibitors. Consequently, the selectivity of NSD2 was notably constrained. Inhibitors targeting the PHD structural domain (NSD2‐PHD) regulate the transcriptional activity of associated proteins by preventing the modification of H3K4me0, H3K4me3, and H3K9me3 by NSD2, but not of H3K36me2.^[^ ^64^, ^65^, ^66^ ^]^ Thus, we opted not to include two classes of inhibitors targeting the SET and PHD structural domains in our selection. Instead we chose UNC6934, an inhibitor targeting the PWWP structural domain, which binds to H3K36me2 via cationic‐π and hydrophobic interactions with the ammonium group of methylated lysines.^[^ ^67^ ^]^ UNC6934 interacts with NSD2‐PWWP1 by occupying the standard methyl‐lysine‐binding pocket, thereby obstructing the interaction between NSD2‐PWWP1 and nuclear H3K36me2. This compound exhibited superior selectivity compared to other inhibitors targeting the PWWP structural domain.^[^ ^42^, ^68^ ^]^ Given the swift advancement of revolutionary technologies and in‐depth exploration of biological functions and mechanisms, a surge in the proposal and development of combinations involving NSD2 inhibitors with other therapeutic agents has been noted. Interestingly, proteolysis‐targeting chimeras against NSD2 have also been introduced.^[^ ^68^ ^]^ We eagerly anticipate the evolution and refinement of these technologies and foresee their future applications in our research domains.

In summary, we elucidated a novel role for the methyltransferase NSD2 in the intestine–liver axis and NASH progression. NSD2 directly regulates the transcriptional activation of ERN1 through the demethylation of histone H3 at lysine 36 (H3K36me2), thereby activating the ERN1–JNK axis to exacerbate intestinal barrier impairment and subsequently promote NASH progression. These findings demonstrate the possibility of discovering intestinal NSD2 inhibitors for the treatment of NASH.

## 4 Experimental Section

### Human Study and Approval

Colon biopsies were obtained from the two cohorts. The patients underwent routine colonoscopy without being diagnosed with NAFLD or NASH. The sex and age were similar between the obese and nonobese groups. IHC staining results and BMI were obtained for cohort 1 (*n* = 40), whereas RT‐PCR, correlation, and western blotting analyses were performed for cohort 2 (*n* = 39). The inclusion and exclusion criteria were as follows: 1) no excessive alcohol consumption (≥140 grams per week for men and ≥70 grams per week for women); 2) no other liver disease (autoimmune hepatitis, hepatitis B or C, etc.); and 3) no medications for hepatoprotective agents or hepatotoxicity in recent years. This study was approved by the Ethics Committee of the 82nd Group Army Hospital of PLA, No. 911, China, and informed consent was obtained from all participants (Approval Number: 2023004).

### Animals

*Nsd2* ^fl/fl^ and Villin‐Cre mice were purchased from ViewSolid Biotech. All mice lines had a C57BL/6J genetic background. Intestine‐specific NSD2 knockout mice were obtained. HFCD was purchased from Qingzilan Technology. Male littermates aged 7–8 weeks were fed either a normal diet (ND) or HFCD for 18 weeks. To generate intestine‐specific NSD2‐overexpressing mice, Lenti‐FLEX‐*Nsd2* (LV‐*Nsd2*) was injected intraperitoneally into Villin‐Cre male mice, and Lenti‐FLEX‐NC control (LV‐NC) was used as a negative control (BGI Genomics Company). The injection dose was 10^9^ viral particles per 200 µL per mouse. Two weeks after the lentivirus injection, the two groups of mice were fed an HFCD for 18 weeks. At week 14, the mice injected with different lentiviruses were randomly divided into two groups, and each group was administered either corn oil (Veh) or UNC6934 (20 mg kg^−1^ body weight dissolved in corn oil) daily. All mice were housed in a temperature‐controlled environment at 20–24 °C, with an average humidity of 40%, following a standard 12‐h light/12‐h dark cycle. They had unlimited access to food and water. At the end of the experiment, the mice were anesthetized, and blood and tissue samples were collected. All experiments using mice were performed per protocols approved by the Animal Research Committee of the China‐Japan Friendship Hospital and the guidelines of the Institute of Clinical Medicine (Approval Number: ZRDWLL0033).

### Total Fecal Excretion and Bomb Calorimetry

Each cage contains no more than three mice. Feces from each cage were collected every three days, dried overnight, and weighed. Finally, the fecal volume per mouse per day was calculated. Fecal energy content was evaluated using a microcalorimeter (Parr) with benzoic acid standards. In summary, six fecal pellets were homogenized in 2 mL of water to form a consistent slurry. The samples were then frozen at −80 °C, and freeze‐drying upon thawing. Each sample was pelletized using a pellet press to create two uniform pellets. Afterward, each pellet underwent individual loading using a microcalorimeter for heat density assessment. The absorbed energy was calculated as the energy ingested in the high‐fat and high‐cholesterol diets each day minus the energy excreted in the feces each day.

### Western Blot

Cell or tissue proteins were extracted using radio immunoprecipitation assay lysis buffer (Beyotime), and nuclear proteins were extracted using commercial kits (Beyotime). The protein concentration was determined using a BCA Protein Assay Kit (Beyotime). Equivalent amounts of protein lysates were separated by 10% polyacrylamide gel electrophoresis and transferred to polyvinylidene difluoride membranes (Millipore). After blocking with 5% bovine serum albumin (BSA), the membrane was incubated overnight at 4 °C with the primary antibody and 1 h at room temperature with a secondary antibody. Signals were detected using an ECL kit (Applygen Technologies Inc.), quantified using ImageJ software, and normalized to β‐actin levels. The antibodies used were as follows: H3K36me2 (A2365, diluted 1:5000, ABclonal), H3K36me3 (ab9050, diluted 1:5000, Abcam), H4K20me2 (ab9052, diluted 1:2000, Abcam), H4K20me3 (A2372, diluted 1:2000, ABclonal), NSD2 (ab75359, diluted 1:5000, Abcam), H3 (AF0009, diluted 1:1000, Beyotime), ZO‐1 (21773‐1‐AP, diluted 1:5000, Proteintech), CLDN1 (13050‐1‐AP, diluted 1:5000, Proteintech), ERN1 (A21021, diluted 1:1000, ABclonal), p‐JNK (80024‐1RR, 1:2000, Proteintech), JNK (66210‐1‐lg, diluted 1:5000, Proteintech), XBP1 (A1731, diluted 1:2000, ABclonal), β‐actin (GB12001‐100, 1:1000, Servicebio).

### Serum Transaminase and Tissue Lipid Assessment

Serum ALT, AST, and TG assays were performed using commercial kits (Elabscience Biotechnology). To evaluate the liver TG and total cholesterol content, the liver tissue was extracted and estimated using commercial kits (Elabscience Biotechnology).

### Intestinal Permeability

Mice were fasted for 12 h, and an intestinal permeability assay was performed using fluorescein isothiocyanate (FITC)‐labeled dextran (MW4000, FD4; Merck). A phosphate‐buffered saline solution containing 600 mg kg^−1^ FD‐4 was administered to the mice. After 4 h of gavage, 100 µL of serum was collected, and the fluorescence intensity was detected using a multifunctional enzyme labeling instrument (Tecan Spark) at an excitation wavelength of 492 nm. Serum FD4 concentrations were obtained from a standard curve based on the fluorescence intensity of the same batch of FD4 measured after serial dilutions. The serum LPS levels were measured using a commercial kit (Cusabio).

### Histology

Fresh liver and intestinal tissues were fixed and cut into frozen or paraffin‐embedded sections. Paraffin slices of liver and intestinal tissues were stained with hematoxylin and eosin (Beyotime). The frozen liver tissue slices were stained with ORO (Solarbio) to observe the accumulation of tissue lipids.

### Glucose and Insulin Tolerance Tests

Mice underwent overnight fasting (16 h) before intraperitoneal administration of glucose at a concentration of 2 g kg^−1^ body weight for GTT. Blood glucose levels were measured in tail clip venous blood using a contour glucometer and contour glucose strips (Bayer) at 0 (baseline), 15, 30, 60, 90, and 120 min after the glucose injection. During the ITT, mice underwent a 5‐h fast during the light cycle before receiving an injection of insulin at a concentration of 0.75 units kg^−1^ body weight. Blood glucose levels were measured in the same manner as in the GTT.

### Immunohistochemistry Staining

IHC was conducted to detect the expression of NSD2 and H3K36me2 in the colon tissues. Paraffin slices were deparaffinized, treated with 0.1% TritonX‐100, and blocked with 3% H_2_O_2_ to inhibit endogenous peroxidase activity, followed by antigen repair. After blocking nonspecific antibody binding with BSA, sections were incubated overnight at 4 °C with primary antibody and then with secondary antibody for 1 h at room temperature. Finally, the slices were stained with DAB, and cell nuclei were labeled with hematoxylin. Images were captured under a light microscope (Olympus) and analyzed using the ImageJ software. Antibodies used were: H3K36me2 (A2365, diluted 1:200, ABclonal), NSD2 (ab75359, diluted 1:150, Abcam).

### Immunofluorescence Staining

Cells or frozen tissue slices were fixed in 4% paraformaldehyde at room temperature and treated with 0.1% Triton‐X 100. After blocking nonspecific antibody binding with BSA, slices were incubated with primary antibody at 4 °C overnight, followed by fluorescent secondary antibody at room temperature for 1 h. Finally, the nuclei were labeled with DAPI (Beyotime). Images were captured using an LSM 780 confocal microscope (Zeiss) and analyzed using ImageJ software. Antibodies used were: F4/80 (28463‐1‐AP, diluted 1:500; Proteintech), ZO‐1 (21773‐1‐AP, diluted 1:300; Proteintech), CLDN1 (13050‐1‐AP, diluted 1:1000; Proteintech), ERN1 (A21021, diluted 1:150; ABclonal).

### Cell Culture and Transfections

Cell lines were acquired from Procell Life Sciences. HT29 cells were cultured in DMEM with 10% fetal bovine serum at 37 °C under 5% CO_2_. Plasmids with NSD2‐specific overexpression (BGI Genomics Company) or shRNA (Public Protein/Plasmid Library) and their corresponding control plasmids were transiently transfected into HT29 cells using Lipofectamine 3000 (Invitrogen). Transfected cells were processed at 6 h post‐transfection and harvested at 48 h post‐transfection unless stated otherwise. Kira6 (1 nM, MCE) treatment of HT29 cells inhibited ERN1 kinase activity. The small‐molecule inhibitor UNC6934 (5 nmol mL^−1^; Selleck Chemicals) was utilized to inhibit the NSD2‐acting protein H3K36me2. Drug‐treated cells were further processed 24 h after administration unless otherwise specified.

### Chromatin Immunoprecipitation (ChIP)

ChIP experiments were conducted using a BeyoChIPTM Enzymatic ChIP Assay Kit (Beyotime). Isotype IgG served as the negative control. The obtained products underwent RT‐PCR. The following antibodies were utilized: H3K36me2 (A2365, diluted 1:2000, ABclonal), IgG (30000‐0‐AP, diluted 1:300, Proteintech), and RNAP2 (A304‐405A, diluted 1:100, Thermo Fisher Scientific). The gene‐specific primers used are listed in Table S3 (Supporting Information).

### Real‐Time qPCR

Total RNA extraction was performed using TRIzol reagent (Invitrogen) according to the manufacturer's instructions, as previously described.^[^ ^70^ ^]^ Gene expression data were normalized to GAPDH as the housekeeping gene. Gene‐specific primers utilized are listed in Table S3 (Supporting Information).

### RNA‐Seq, CUT&Tag‐Seq, and Data Analysis

RNA‐Seq was conducted using the HyperactiveTM in situ ChIP Library Prep Kit for Illumina (Vazyme Biotech), following the manufacturer's instructions for the CUT&Tag assay.^[^ ^69^ ^]^ Briefly, the H3K36me2 antibody directed the ChiTag enzyme to the chromatin localized by the H3K36me2 protein, resulting in DNA fragments. During the enzymatic reaction, the ChiTag enzyme attaches specific sequencing junctions to newly generated DNA fragments, and accordingly, the labeled DNA fragments were released directly from the nucleus of the cell. Subsequent PCR amplification using specific primers yielded purified PCR products that were evaluated using an Agilent 2100 Bioanalyzer (Agilent Technologies). These libraries were then sequenced and subsequently analyzed.

### Statistical Analysis

Unless otherwise specified, data were processed using GraphPad Prism 9 for statistical analysis. All biological assay data were presented as the mean ± SEM. For comparisons between the two groups, significance was determined using a two‐tailed Student's t‐test. For comparisons involving more than two groups, one‐way analysis of variance followed by post hoc Bonferroni tests was performed. Differences were considered statistically significant at p < 0.05.

## Conflict of Interest

The authors declare no conflict of interest.

## Supporting information

Supporting Information

## Data Availability

The RNA‐Seq and CUT&Tag data are deposited in the Gene Expression Omnibus (GSE254667; GSE254668).
