# Supplementary material for: Intestinal NSD2 Aggravates Nonalcoholic Steatohepatitis Through Histone Modifications
Source: Adv Sci (Weinh). 2024 Jun 26;11(33):2402551. doi: 10.1002/advs.202402551 (PMC11434126; doi:10.1002/advs.202402551)
Supplement: Supplementary file 1 — Supporting Information [file ADVS-11-2402551-s001.docx]

Supporting Information

Intestinal NSD2 aggravates nonalcoholic steatohepatitis through histone modifications

Yijia Zhang, Yuan Qiao, Zecheng Li, Donghai Liu, Qi Jin, Jing Guo, Xin Li, Long Chen*, Lihong Liu*, and Liang Peng*

Figures S1-6

Tables S1-3

Supporting Information

Figures S1-6


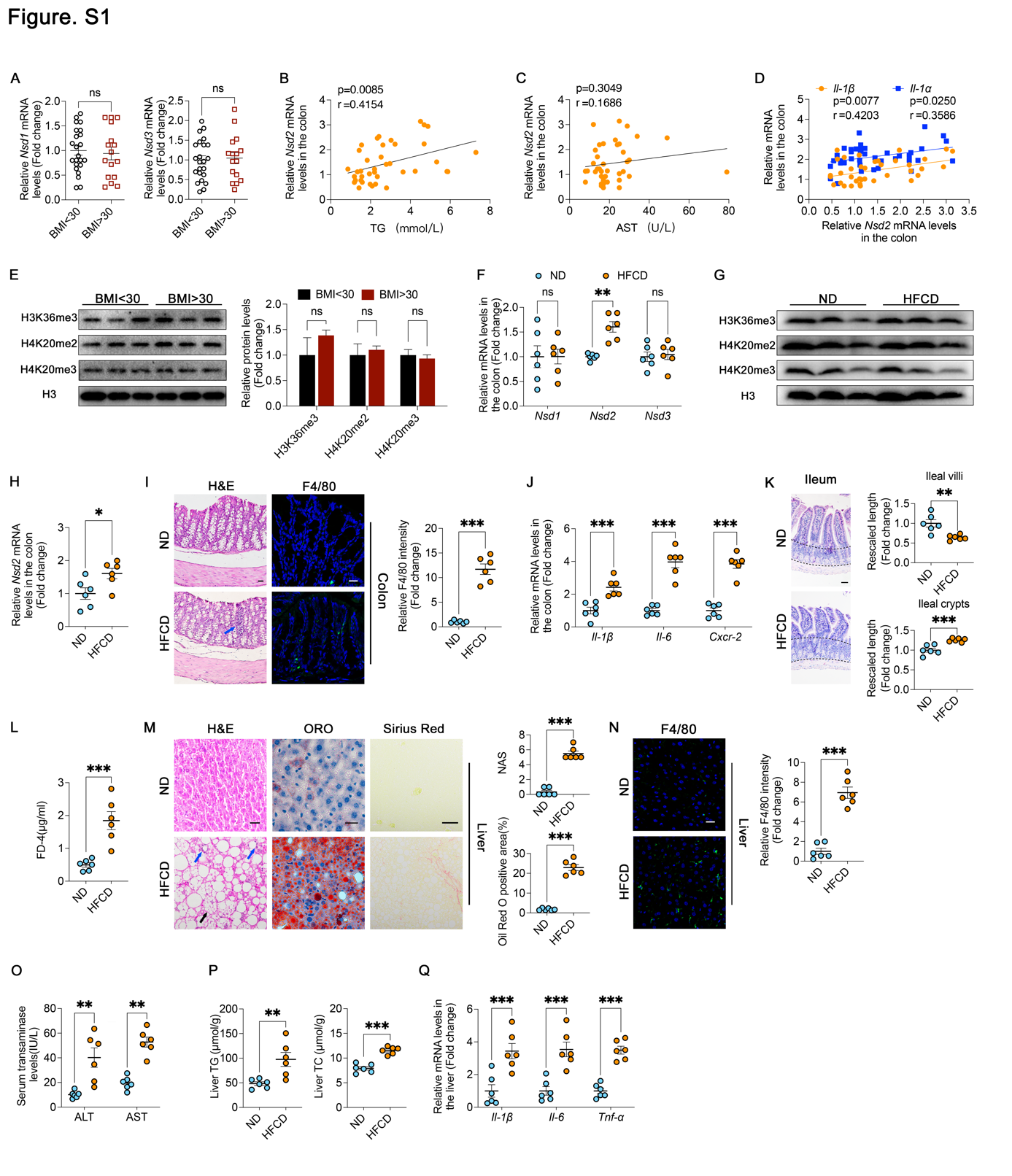


**Figure S1. Intestinal NSD2 and H3K36me2 levels are increased in humans with obesity and HFCD-fed mice.** A) mRNA expression levels of NSDs in human colon biopsies from individuals without obesity (n=23, cohort 2) and with obesity (n=16, cohort 2). B-D) Two-tailed Pearson’s correlation coefficient analysis of colon biopsy *Nsd2* levels with serum TG levels (B), serum AST level (C), and mRNA levels of colon biopsy *Il-1α* and *Il-1β* (D), respectively (cohort 2). E) Proteins levels of colon biopsy H3K36me3, H4K20me2 and H4K20me3 (cohort 2). F) mRNA expression levels of *Nsd* family in the mice colon (n=6). G) Representative proteins expression levels of H3K36me3, H4K20me2 and H4K20me3 in the colon of ND- or HFCD- fed mice (n=3). H) mRNA expression levels of *Nsd*2 in colon of mice fed with ND or HFCD for two weeks (n=6). I) Representative H&E staining (left) and immunofluorescence staining of F4/80 (right) of colon sections (n = 6). Scale bars, 20µm. J) Relative mRNA levels of *Il-1β* and *Il-6* and *Cxcr2* in the mice colon (n=6). K) H&E staining of ileum (left), and lengths of ileal villi and crypts as indicated (right, n=6 villi or crypts). Scale bars, 50µm. L) Intestinal permeability assay. (n=6). M) Representative H&E staining (left), Oil Red O staining (middle) and Sirius red staining (right) of liver sections from ND or HFCD fed mice. Blue arrows indicate inflammatory cell infiltration and black arrows indicate ballooning degeneration in hepatocytes (n=6). Scale bars, 20µm (H&E staining and Oil Red O staining) or 50µm (Sirius red staining). N) Representative immunofluorescence staining of F4/80 (green) and DAPI (blue) in mice liver (n=6). Scale bars, 20µm. O) Serum ALT and AST concentrations in the indicated groups of mice (n=6). P) Serum TG (left) and TC (right) concentrations in the indicated groups of mice (n=6). Q) Relative mRNA levels of *Il-1β* and *Il-6* and *Tnf-α* in the mice liver (n=6). The results are presented as means ± SEM, *p <0.05, **p < 0.01, ***p < 0.001. ns means not significant. Statistical analyses were performed by two-tailed t-tests between two groups, unless otherwise stated.


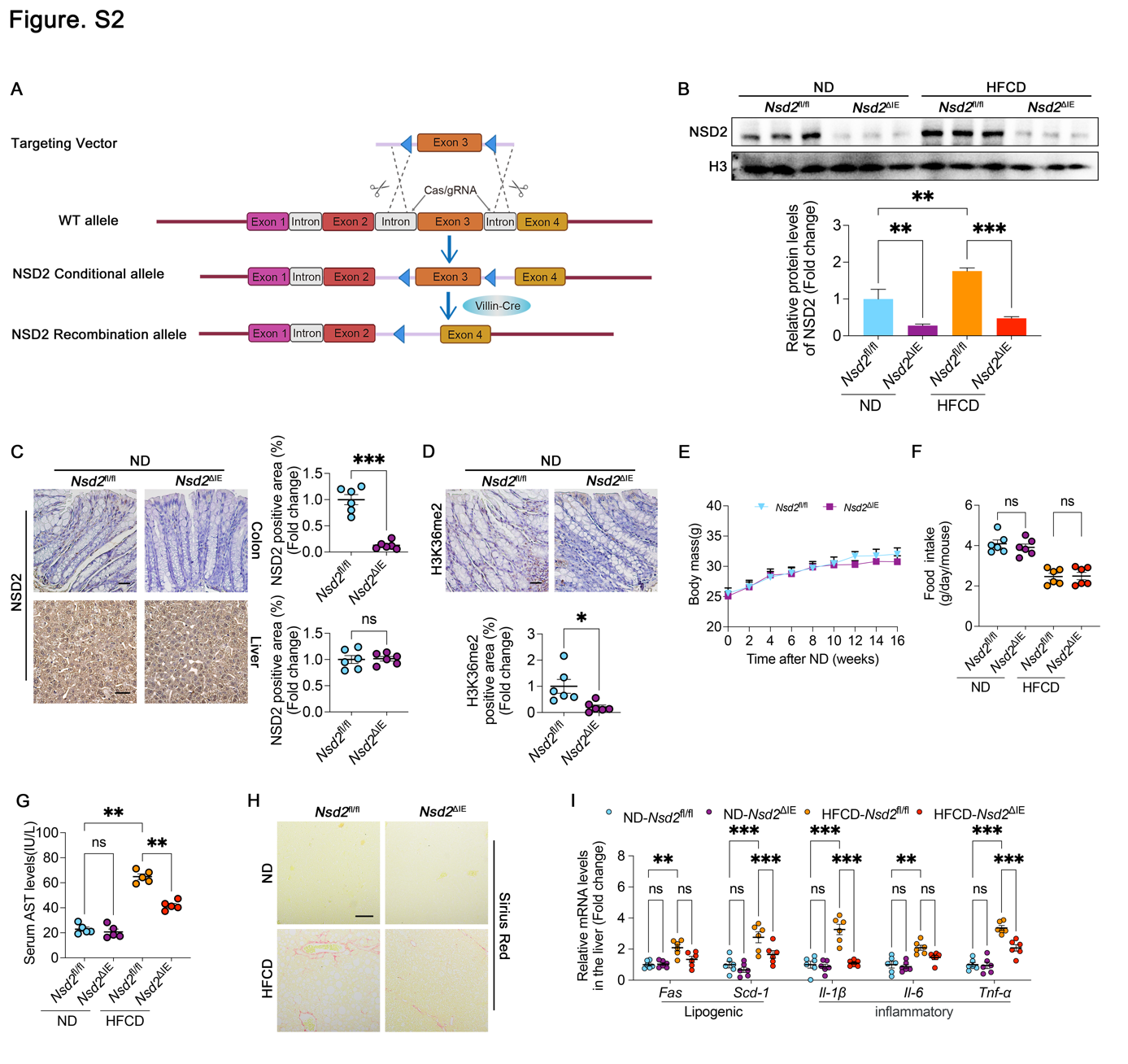


**Figure S2.** **Intestine-specific NSD2 knockout attenuates NASH profiles.** A) Generation of an intestine-specific knockout mouse model of the *Nsd2* gene. B) Representative protein expression level (n=3) in the ileum of indicated mice groups. C) IHC staining for the expression of NSD2 (left) and quantification (right) in colon and in liver of indicated mice groups (n=6). Scale bar, 20µm. D) IHC staining for the expression of H3K36me2 in colon of indicated mice groups (n=6). Scale bar, 20µm. E, F) Growth curves of ND-fed *Nsd2*^fl/fl^ and *Nsd2*^∆IE^ mice (E) and food intake per day (F) of ND or HFCD-fed *Nsd2*^fl/fl^ and *Nsd2*^∆IE^ mice (n=6). G) Serum AST concentrations in the indicated groups of mice (n=6). H) Representative Sirius red staining of liver sections (n=6). Scale bars, 50µm. I) Adipogenic genes and proinflammatory cytokines related mRNA levels. (n=6). The results are presented as means ± SEM, *p <0.05, **p < 0.01, ***p < 0.001. ns means not significant. Statistical analyses were performed by two-tailed t-tests between two groups, while one-way ANOVA and post hoc Bonferroni tests were performed between multiple groups.


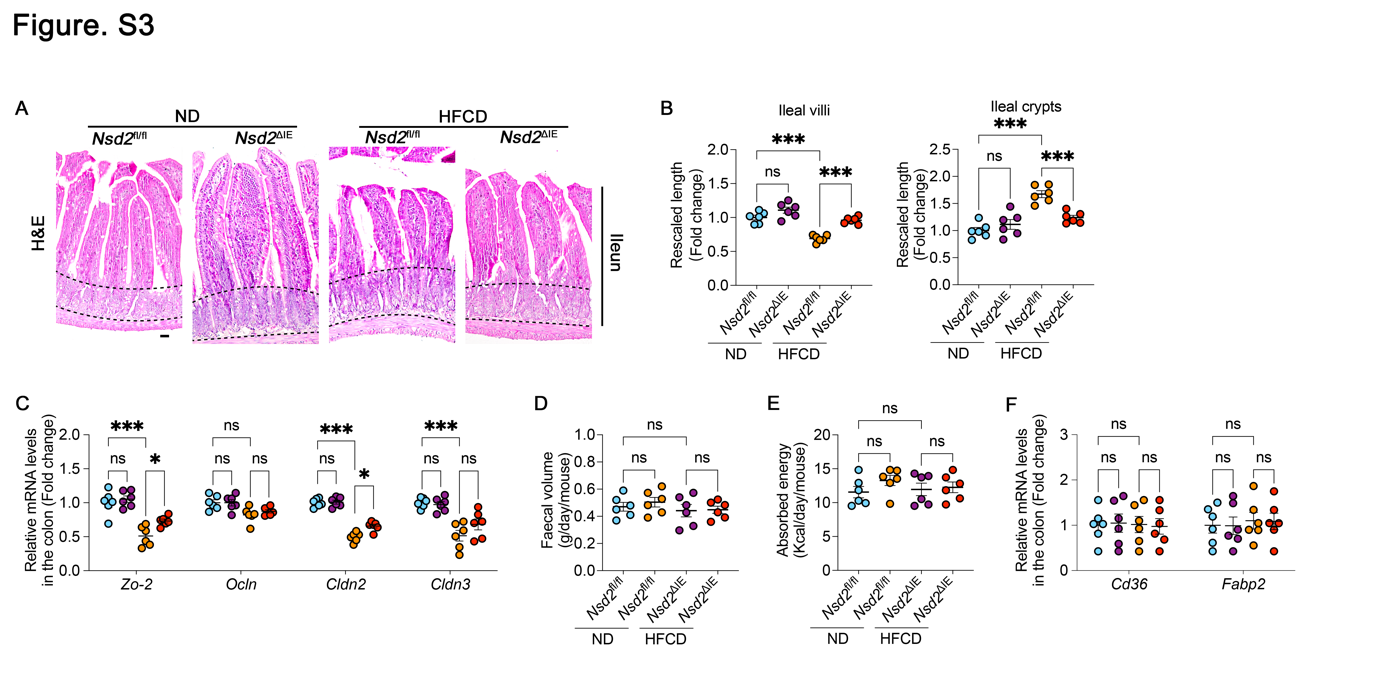


**Figure S3. Intestine-specific knockout protects mice from HFCD-induced intestinal epithelial barrier impairment.** A, B) H&E staining of ileum from *Nsd2*^fl/fl^ and *Nsd2*^∆IE^ mice fed ND or HFCD (A), and lengths of ileal villi and crypts as indicated (n=6 villi or crypts; B). C) Relative mRNA levels of intestinal barrier (*Zo-2, Ocln, Cldn2* and *Cldn3*) in colon of *Nsd2*^fl/fl^ and *Nsd2*^∆IE^ mice fed HFCD or ND (n=6). D) Daily fecal excretion in *Nsd2*^fl/fl^ and *Nsd2*^∆IE^ mice fed ND or HFCD. E) Energy absorption in *Nsd2*^fl/fl^ and *Nsd2*^∆IE^ mice fed ND or HFCD (n=6). Absorbed energy calculated from food intake in kilocalories per day minus residual fecal energy in kilocalories per day. F) Relative mRNA levels of intestinal lipid absorption (*Cd36, Fabp2*) in colon of *Nsd2*^fl/fl^ and *Nsd2*^∆IE^ mice fed HFCD or ND (n=6). The results are presented as means ± SEM, *p <0.05, **p < 0.01, ***p < 0.001. ns means not significant. Statistical analyses were performed one-way ANOVA and post hoc Bonferroni tests.


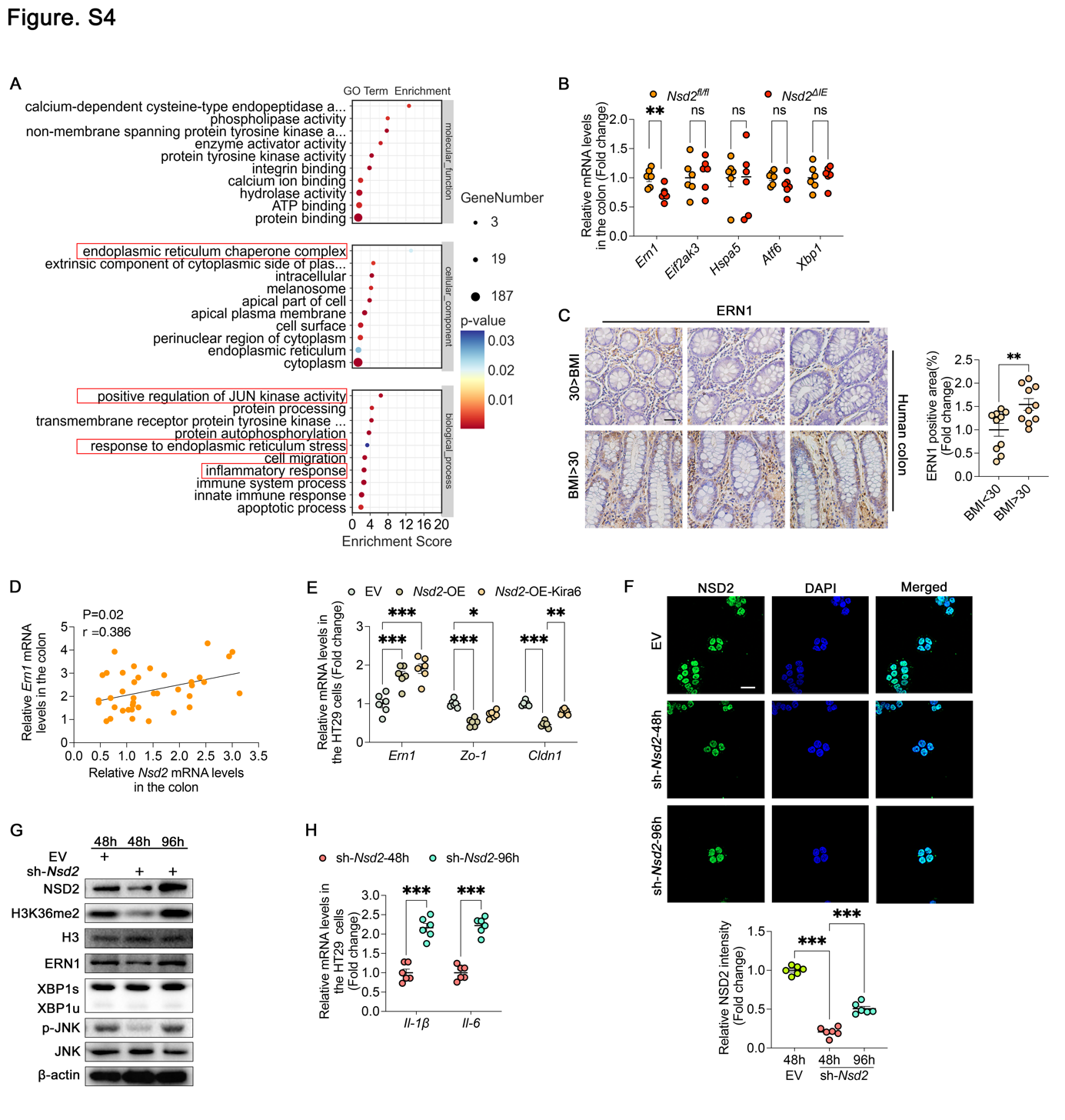


**Figure S4. NSD2 deficiency dampens the ERN1–JNK axis in the intestine of HFCD-fed mice.** A) GO analyses show the altered pathways after NSD2 knockout. B) Relative mRNA levels of ER stress sensors (*Ern1*, *Eif2ak3*, *Hspa5,* *Atf6*, and *Xbp1*) in colon of *Nsd2*^fl/fl^ and *Nsd2*^∆IE^ mice fed HFCD (n=6). C) IHC staining for the expression of ERN1 in human colon biopsies from cohort 1 (n=10 subjects/group). Scale bar, 20μm. D) Two-tailed Pearson’s correlation coefficient analysis of colon biopsy *Nsd2* levels with mRNA levels of colon biopsy *Ern1* (cohort 2). E) Relative mRNA levels in HT29 of different groups (n=6). F) Immunofluorescence staining of NSD2 in HT29 cells. Scale bar, 5μm. G) Representative proteins expression levels in the HT29 cells. H) Relative mRNA levels of *Il-1β* and *Il-6* in HT29 of different groups (n=6). The results are presented as means ± SEM, *p <0.05, **p < 0.01, ***p < 0.001. ns means not significant. Statistical analyses were performed by two-tailed t-tests between two groups, while one-way ANOVA and post hoc Bonferroni tests were performed between multiple groups.


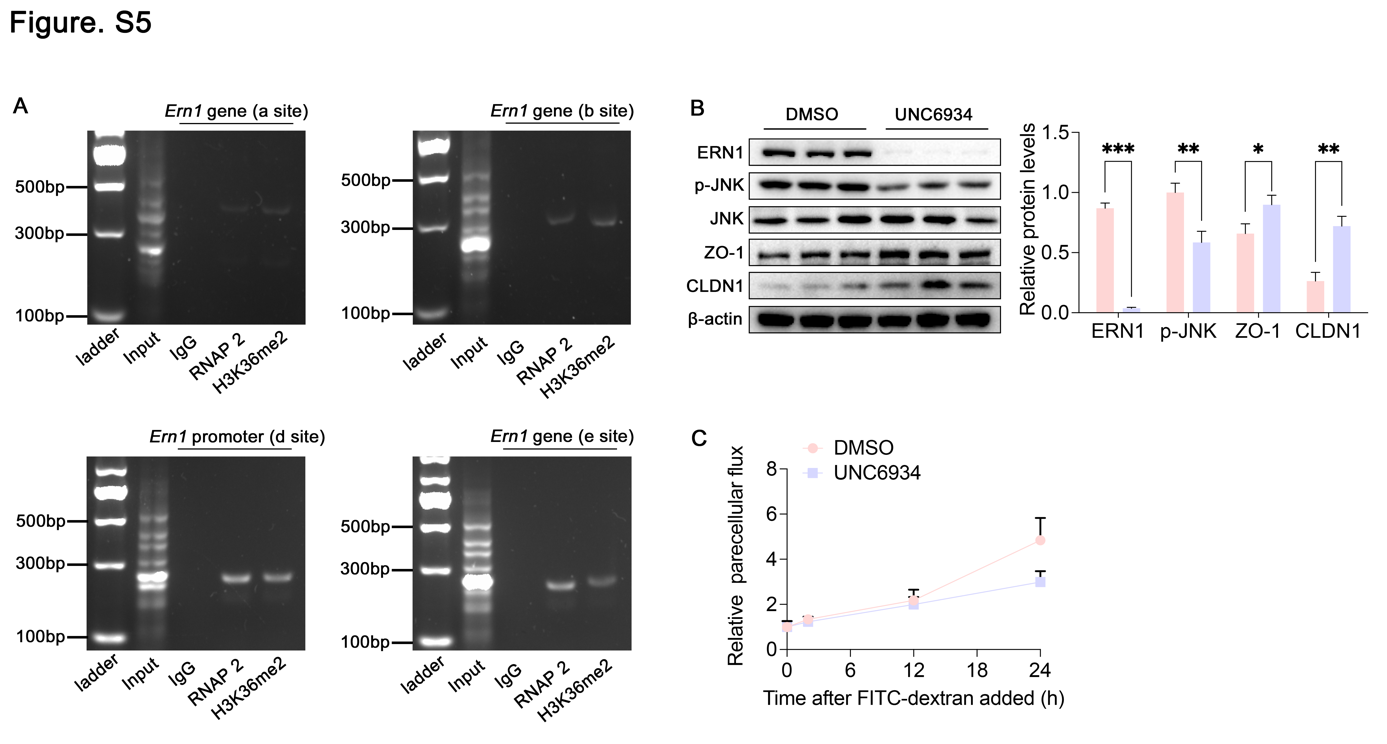


**Figure S5. Histone methyltransferase activity of NSD2 mediates the upregulation of ERN1.** A) ChIP-PCR analysis to detect H3K36me2 binding in the *Ern1* promoter and nearby regions. B) Representative proteins expression levels (n=3) in HT29 cells treated with or without UNC6934. C) Epithelial FITC-dextran leakage assay in two groups of HT29 cells, DMSO and UNC6934 (n=6). The results are presented as means ± SEM, *p <0.05, **p < 0.01, ***p < 0.001. ns means not significant. Statistical analyses were performed by two-tailed t-tests.


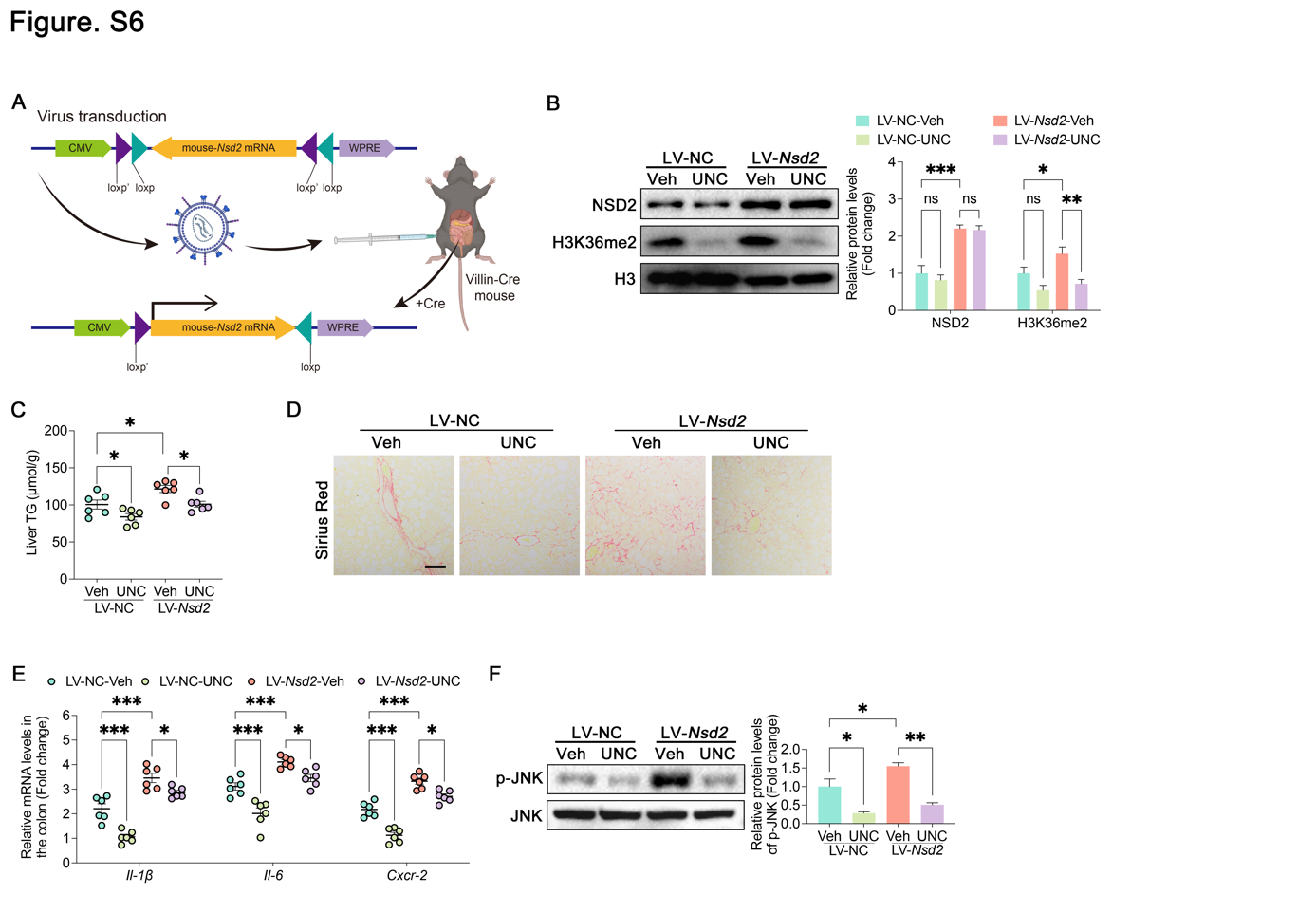


**Figure S6. Intestine-specific NSD2 overexpression aggravated the HFCD-induced NASH phenotype through histone modifications.** A) LV-*Nsd2* injected in a Villin-Cre mouse. B) Representative proteins expression levels in the colon of indicated mice groups. (n=3). C) Liver TG levels in the indicated groups of mice (n=6). D) Representative Sirius red staining of liver sections (n=6). Scale bars, 50µm. E) Relative mRNA levels of proinflammatory cytokines in the colon of indicated mice groups (n=6). F) Representative protein expression level of p-JNK in the colon of indicated mice groups (n=3). The results are presented as means ± SEM, *p <0.05, **p < 0.01, ***p < 0.001. ns means not significant. Statistical analyses were performed by one-way ANOVA and post hoc Bonferroni tests were performed between multiple groups.

**Tables S1-3**

**Table S1.** Clinical sample information of cohort 1.

| Number | Gender | Age  (years) | Height  (m) | Weight  (kg) | BMI  (kg/m^2^) |
| --- | --- | --- | --- | --- | --- |
| A1 | Female | 58 | 1.65 | 45 | 16.5 |
| A2 | Male | 62 | 1.72 | 55 | 18.6 |
| A3 | Female | 49 | 1.55 | 45 | 18.7 |
| A4 | Male | 70 | 1.63 | 50 | 18.8 |
| A5 | Male | 50 | 1.74 | 59 | 19.5 |
| A6 | Male | 30 | 1.65 | 55 | 20.2 |
| A7 | Female | 58 | 1.63 | 57 | 21.5 |
| A8 | Male | 67 | 1.83 | 72 | 21.5 |
| A9 | Male | 72 | 1.79 | 69 | 21.5 |
| A10 | Female | 69 | 1.59 | 55 | 21.8 |
| A11 | Male | 48 | 1.80 | 82 | 25.3 |
| A12 | Female | 66 | 1.65 | 69 | 25.3 |
| A13 | Male | 64 | 1.68 | 72 | 25.5 |
| A14 | Male | 51 | 1.63 | 68 | 25.6 |
| A15 | Male | 58 | 1.76 | 80 | 25.8 |
| A16 | Female | 69 | 1.52 | 60 | 26.0 |
| A17 | Female | 67 | 1.55 | 63 | 26.2 |
| A18 | Male | 66 | 1.75 | 64 | 27.4 |
| A19 | Male | 37 | 1.70 | 81 | 28.0 |
| A20 | Female | 55 | 1.60 | 72 | 28.1 |
| A21 | Female | 54 | 1.60 | 77 | 30.1 |
| A22 | Male | 42 | 1.85 | 103 | 30.1 |
| A23 | Female | 47 | 1.70 | 87 | 30.1 |
| A24 | Male | 55 | 1.79 | 98 | 30.6 |
| A25 | Female | 46 | 1.71 | 90 | 30.8 |
| A26 | Male | 35 | 1.62 | 81 | 30.9 |
| A27 | Female | 35 | 1.75 | 95 | 31.0 |
| A28 | Male | 39 | 1.86 | 110 | 31.8 |
| A29 | Female | 47 | 1.55 | 79 | 32.9 |
| A30 | Male | 63 | 1.74 | 102 | 33.7 |
| A31 | Male | 26 | 1.84 | 120 | 35.4 |
| A32 | Female | 39 | 1.63 | 95 | 35.8 |
| A33 | Male | 33 | 1.79 | 115 | 35.9 |
| A34 | Female | 35 | 1.65 | 98 | 36.0 |
| A35 | Female | 38 | 1.65 | 98 | 35.6 |
| A36 | Female | 59 | 1.55 | 88 | 36.6 |
| A37 | Female | 49 | 1.64 | 99 | 36.8 |
| A38 | Male | 56 | 1.82 | 124 | 37.4 |
| A39 | Male | 65 | 1.87 | 137 | 39.2 |
| A40 | Male | 65 | 1.79 | 127.5 | 39.8 |

**Table S2.** Clinical sample information of cohort 2.

| Number | Gender | Age  (years) | Height  (m) | Weight  (kg) | BMI  (kg/m^2^) | ALT  (U/L) | AST  (U/L) | TG  (mmol/L) |
| --- | --- | --- | --- | --- | --- | --- | --- | --- |
| B1 | Female | 62 | 1.58 | 55 | 22.0 | 15 | 27 | 7.29 |
| B2 | Male | 61 | 1.74 | 70 | 23.1 | 27 | 15 | 2.20 |
| B3 | Female | 51 | 1.60 | 61 | 23.8 | 12 | 19 | 1.58 |
| B4 | Female | 55 | 1.58 | 60 | 24.0 | 8 | 17 | 1.24 |
| B5 | Female | 58 | 1.70 | 70 | 24.2 | 34 | 25 | 2.80 |
| B6 | Male | 80 | 1.70 | 70 | 24.2 | 13 | 17 | 1.42 |
| B7 | Male | 65 | 1.72 | 72 | 24.3 | 25 | 18 | 2.34 |
| B8 | Female | 26 | 1.75 | 79 | 25.8 | 25 | 19 | 1.51 |
| B9 | Male | 65 | 1.70 | 75 | 26.0 | 20 | 17 | 2.79 |
| B10 | Male | 32 | 1.75 | 82 | 26.8 | 67 | 30 | 1.83 |
| B11 | Female | 35 | 1.70 | 80 | 27.7 | 100 | 79 | 2.20 |
| B12 | Male | 67 | 1.64 | 65 | 24.2 | 11 | 13 | 3.63 |
| B13 | Female | 66 | 1.60 | 62 | 24.2 | 30 | 27 | 1.34 |
| B14 | Female | 48 | 1.72 | 73 | 24.7 | 21 | 16 | 5.88 |
| B15 | Female | 76 | 1.69 | 74 | 25.9 | 13 | 8 | 5.84 |
| B16 | Male | 67 | 1.71 | 78 | 26.7 | 16 | 9 | 4.24 |
| B17 | Female | 70 | 1.58 | 70 | 28.0 | 39 | 30 | 1.56 |
| B18 | Female | 57 | 1.65 | 77 | 28.3 | 13 | 8 | 1.19 |
| B19 | Female | 67 | 1.57 | 70 | 28.4 | 20 | 14 | 0.86 |
| B20 | Male | 55 | 1.75 | 87.5 | 28.6 | 20 | 17 | 1.98 |
| B21 | Male | 44 | 1.72 | 85 | 28.7 | 21 | 16 | 1.19 |
| B22 | Male | 65 | 1.76 | 90 | 29.1 | 35 | 25 | 1.52 |
| B23 | Male | 63 | 1.70 | 85 | 29.4 | 17 | 16 | 2.08 |
| B24 | Male | 49 | 1.72 | 89 | 30.1 | 71 | 34 | 2.42 |
| B25 | Female | 49 | 1.60 | 78 | 30.47 | 33 | 25 | 1.94 |
| B26 | Male | 40 | 1.80 | 99 | 30.56 | 32 | 12 | 2.97 |
| B27 | Female | 55 | 1.67 | 87 | 31.2 | 32 | 14 | 2.53 |
| B28 | Male | 42 | 1.72 | 96 | 32.4 | 18 | 13 | 1.43 |
| B29 | Male | 50 | 1.75 | 105 | 34.3 | 25 | 17 | 4.69 |
| B30 | Male | 23 | 1.81 | 120 | 36.6 | 28 | 49 | 2.34 |
| B31 | Female | 53 | 1.62 | 90 | 34.3 | 45 | 12 | 4.69 |
| B32 | Female | 55 | 1.60 | 86 | 33.6 | 29 | 25 | 4.51 |
| B33 | Female | 32 | 1.68 | 95 | 33.7 | 33 | 16 | 2.25 |
| B34 | Male | 53 | 1.83 | 131 | 39.1 | 37 | 29 | 5.32 |
| B35 | Female | 37 | 1.76 | 96 | 31.0 | 20 | 15 | 1.55 |
| B36 | Male | 62 | 1.79 | 135 | 42.1 | 37 | 24 | 2.62 |
| B37 | Male | 65 | 1.75 | 116 | 37.9 | 34 | 25 | 3.23 |
| B38 | Male | 45 | 1.80 | 137 | 42.3 | 39 | 23 | 3.03 |
| B39 | Female | 43 | 1.69 | 102 | 35.7 | 35 | 29 | 4.88 |

**Table S3.** PCR primer sequences of related genes.

| GENE | Forward  primer (5’-3’) | Reverse  primer (5’-3’) |
| --- | --- | --- |
| h*-Nsd1* | TCCTGAGTCAGAACATGACCTG | CGAGATTTAGCGCAAGGCTTTT |
| h*-Nsd2* | ACCGCGAGTGTTCTGTGTTC | GTCGTGGCCGTTAAACTTCTG |
| h*-Nsd3* | AACTCATTGACTCCGCCAACA | CTGAAAGCCTTGCTGCAAAGT |
| h*-Il-1β* | ATGATGGCTTATTACAGTGGCAA | GTCGGAGATTCGTAGCTGGA |
| h*-IL-1α* | AGATGCCTGAGATACCCAAAACC | CCAAGCACACCCAGTAGTCT |
| h*-Zo-1* | CAACATACAGTGACGCTTCACA | CACTATTGACGTTTCCCCACTC |
| h*-Cldn1* | CCTCCTGGGAGTGATAGCAAT | GGCAACTAAAATAGCCAGACCT |
| h*-Ern1* | CACAGTGACGCTTCCTGAAAC | GCCATCATTAGGATCTGGGAGA |
| h*-Gapdh* | TGAAGGTCGGAGTCAACGGA | CCATTGATGACAAGCTTCCCG |
| m*-Nsd2* | TGCCAAAAAGGAGTACGTGTG | CTTCGGGAAAGTCCAAGGCAG |
| m*-Il-1β* | GAAATGCCACCTTTTGACAGTG | TGGATGCTCTCATCAGGACAG |
| m*-Il6* | CCAAGAGGTGAGTGCTTCCC | CTGTTGTTCAGACTCTCTCCCT |
| m*-Tnf-α* | CCCTCACACTCAGATCATCTTCT | GCTACGACGTGGGCTACAG |
| m*-Fas* | GGAGGTGGTGATAGCCGGTAT | TGGGTAATCCATAGAGCCCAG |
| m*-Scd-1* | TTCTTGCGATACACTCTGGTGC | CGGGATTGAATGTTCTTGTCGT |
| m*-Cxcr2* | ATGCCCTCTATTCTGCCAGAT | GTGCTCCGGTTGTATAAGATGAC |
| m*-Zo-1* | ACCACCAACCCGAGAAGAC | CAGGAGTCATGGACGCACA |
| m-*Zo-2* | ATGGGAGCAGTACACCGTGA | TGACCACCCTGTCATTTTCTTG |
| m-*Ocln* | TGAAAGTCCACCTCCTTACAGA | CCGGATAAAAAGAGTACGCTGG |
| m*-Cldn1* | GGGGACAACATCGTGACCG | AGGAGTCGAAGACTTTGCACT |
| m*-Cldn2* | CAACTGGTGGGCTACATCCTA | CCCTTGGAAAAGCCAACCG |
| m*-Cldn3* | ACCAACTGCGTACAAGACGAG | CAGAGCCGCCAACAGGAAA |
| m*-Gapdh* | CCCCAATGTGTCCGTCGTG | GCCTGCTTCACCACCTTCT |
| m-Ch-*Ern1*(a) | TGGATTTTTCCCTTGCCTTTGGGGC | TAAAGCTGAAGCCACAGCGCA |
| m-Ch-*Ern1*(b) | ACTCGGAGCTGTCTCCCAAA | ATTCCCGGCTTCAAACGCTT |
| m-Ch-*Ern1*(c) | TGAGTGCAGATGCCCCTAGAGT | ATACGCAAACCCCCACTCATACTC |
| m-Ch-*Ern1*(d) | CGAAAGGGCTGACAAGATGGTTC | ACACCATCAGCCAGTGACTTGG |
| m-Ch-*Ern1*(e) | CGATAGCCCAGGGGAAAACAAG | TAGTTGCCAGCTGGCTGAGGA |
| h-Ch-*Ern1*(a) | GTCACCGAGAACTGAGGATC | TGCGGCTCACTAGATTTATGT |
| h-Ch-*Ern1*(b) | GCGCTTCGAATCCTTGTTTGA | CAGCTGAGTGAAGGTCATAAAGAG |
| h-Ch-*Ern1*(c) | GGTGGTGAGTGATAGGTAGCG | CCCTTTACCACAGAATCCCACT |
| h-Ch-*Ern1*(d) | TACTTCCACACACTCTGTAGGTTCA | AAAAAAGGAGCTTGTGCCTGTC |
| h-Ch-*Ern1*(e) | CCCGGCAGTGTGTTTTAATT | GGTTCCTCTGGGTTTTCAAG |

Primers beginning with "h" indicate that they are used for detection of clinical samples or HT29 cells. Primers beginning with "m" mean that they are used for detection of mouse tissue. "Ch" mean that Primers are used for ChIP-qPCR.
